# The use of the CHAID algorithm for determining tourism segmentation: A purposeful outcome^☆^

**DOI:** 10.1016/j.heliyon.2020.e04256

**Published:** 2020-07-08

**Authors:** Flora Ma Díaz-Pérez, Carlos G. García-González, Alan Fyall

**Affiliations:** a Facultad de Economía, Empresa y Turismo, Universidad de La Laguna, 38071, La Laguna-Tenerife, Spain; b Rosen College of Hospitality Management, University of Central Florida, 9907 Universal Boulevard, Orlando, FL 32819, USA

**Keywords:** Tourism market segmentation, CHAID algorithm, Means of transportation used, Canary resident/non-residents, Economic impacts, Environmental impacts, Accommodation, Stays, Statistics, Tourism, Environmental economics, Tourism economics, Marketing

## Abstract

This paper considers the most suitable market segment(s) from an environmental and local economic development perspective in the specific context of visits to natural environments. More specifically, the paper explores the distinctions and differences between tourists (non-residents) and residents with regard to visit behavior at natural attractions. By using the CHAID algorithm, a decision tree is constructed for means of transportation which serves as a key factor in the segmentation process. However, such a tree for visitors' resident or non-resident status cannot be built as a first explicative variable, unless it is statistically forced. Once it is forced, the tree opens in several sub-segments, for non-residents and residents alike. Finally, it allows understanding of the means of transportation used by visitors according to their geographical origin as well as a set of added independent variables: accommodation establishment, length of stay, season, and other demographic variables (educational level, gender, and age). Also, more importantly, we have obtained segments with no overlap configured according to all the aforementioned variables. This is a very strong result from a methodological point of view and for policy makers in destination settings.

## 1 Introduction

In the specific context of tourism marketing, clustering the population of visitors to a natural attraction is a fundamental strategy to understand real tourism market behavior and impacts. A number of studies have thus confirmed the need to examine market segmentation practices more deeply in order to ensure the segmentation of markets is as accurate and as meaningful as possible (Cook and Mindak, 1984; Kardes, 2002; Mok and Iverson, 2000; Rhim and Cooper, 2005; Solomon et al., 2002). In this regard, Dolnicar et al. (2014) says “In the tourism industry, many key strategic decisions are made based on market segmentation studies, including the choice of a target segment and the development of an entire marketing mix to suit this segment” (p.296). In spite of that**,** segmentation studies relating to tourism destinations have received less prominence than the analysis of a particular company situation. Much of this can be attributed to difficulty in obtaining aggregated data in the destination context (Bowen, 1998; Chung et al., 2004; Dixona et al., 2012; Koc and Altinay, 2007; Mak, 2004; Pizam and Reichel, 1979; Snowball, 2004; Spotts and Mahoney, 1991).

Consequently, identifying the most appropriate and efficient methods to investigate how different market segments affect destinations is a crucial decision. There has been a range of segmentation techniques used, the Chi-square Automatic Interaction Detection (CHAID) algorithm being one of the most effective. The primary advantage of this technique is the large number of variables that can be used in the segmentation process and how it offers destination operators a complete market study for a specific objective using a particular criterion variable (Díaz-Pérez and Bethencourt-Cejas, 2016). Nevertheless the literature on tourism market segmentation has shown very little interest in non-parametric models such as CHAID. Rather, the focus has been on parametric models based on the restriction of normal distributions. However, progress in computer science and the use of algorithms has made possible the application of non-parametric statistics. This in turn has opened up a realm of possibilities for the analysis of tourism markets. In particular, it has allowed the researcher to obtain an important number of market clusters. Interestingly, this procedure has been used frequently in the domain of health studies (Godley et al., 1998; Forthofer and Bryant, 2000; Sullivan and van Zyl, 2008; Peralta Dela Cruz, 2018), but not with such intensity in the context of tourism market segmentation.

Traditionally, demographic variables have been used to identify the most relevant market segments (see for example, Koc, 2002; Koc, 2004; Nicholson and Pearce, 2000; Shoemaker, 1984; Shoemaker, 1989). In this respect, the geographic origin of tourists has been studied as one of the primary variables of segmentation (Hsu and Kang, 2007; Koc and Altinay, 2007; Legohérel et al., 2015; Ng and Lew, 2009). Interestingly, very few such studies breakdown the market by domestic and international visitor origin (see Erdilek and Wolf, 1997; Hadjikakou et al., 2014; Thrane and Farstad, 2012). This is even less so when the CHAID algorithm is used as a basis of segmentation (Amir et al., 2016). Although Díaz-Pérez and Bethencourt-Cejas (2017) tried to incorporate into their analysis the variable resident/non-resident to segment the visitors' population to the Teide National Park, the results obtained were not successful for that time. This was explained by the decision tree obtained not opening for this geographical variable. Moreover the previous study did not use as the dependent variable the mean of transportation.

However, for the tourism industry, it is particularly relevant when the measurement of the impact on the local resources is based on the means of transportation used (Gossling et al., 2009; Page and Connell, 2009). Transportation in tourism development is recognized as a strategic sector (Amir et al., 2016; Middleton, 1998; Reilly et al., 2010). It assists tourist movement (Masiero and Zoltan, 2013), provides employment to the local community (Williams and Ponsford, 2009), as well as enhances commercial and business activity by providing the required connectivity (Leiper, 2004; Lew and Mckercher, 2006). Therefore, transportation has been given much emphasis in government planning policies as it is a major influence on any tourist destination (Gatta et al., 2017; Peeters and Schouten, 2006).

Although transportation has received much prominence in government planning policies and the academic literature, its contribution to studies on market segmentation is, however, rare; this being hard to fathom due to its major contribution to the dynamics of any tourist destination (see for example the study on Amsterdam by Peeters and Schouten, 2006). In light of the significance of accommodation type, duration of the visit, and the geographic origin of tourists along with other demographic variables, it really is surprising to see such a vacuum of studies that bring these important variables together for purposes of market segmentation. Some papers have used the CHAID algorithm as the analytical method with the study by McKercher and Tse (2012) looking at intention to return as a proxy for actual repeat visitation. Other studies, meanwhile, have considered transport choice behavior at destinations (Robbins and Thompson, 2007) and travel choice mode (Nerhagan, 2003; Koppelman and Sethi, 2005; Tsuar and Wu, 2005). None, however, have studied the relationship between mode of transportation and tourists' geographic origins in the context of segmentation.

This paper takes into consideration the above discussion which serves as a platform for the study of the visitors' population to the Teide National Park, in Canary Islands (Spain).

### 1.1 The impacts of the visitors to the Teide National Park (TNP)

Problems arising from the negative impacts of the number of visitors on parks' natural resources are undoubtedly a challenge for a destination's long-term sustainability. The Canary Islands, a Spanish autonomous community, consists of several islands and contains four of the fifteen Spanish National Parks (NPs). In fact, the Canary Islands NPs have a total amount of visitors which is nearly half the total number of visitors to Spanish parks as a whole (ISTAC, 2017). Considering that the total number of visitors to a NP could be characterized by being segmented in different groups, the first step in this analysis focused on El Teide National Park (island of Tenerife) will be segmenting the complete group to determine which of these particular segments generates the greatest environmental impact. At the same time, and taking into account the important economic contribution of touristic activity to the Canary Islands Gross Domestic Product (ISTAC, 2017), the incomes generated from visits would be considered as a relevant issue in the segmentation process.

In this paper, correlations between means of transportation used by tourists and geographic origin are studied first. Ultimately, this study seeks to address the following question: is there any relevant segmentation between Canary Islands resident and non-resident visitors to national parks? And also, which is the role played by the accommodation establishment chosen by the visitors for these different geographical origins? Are there any other significant variables influencing the means of transportation used by the visitor to get to the TNP? Is the CHAID algorithm an appropriate method of determining market segmentation when segmentation is based on the above dependent/independent variables?

Considering all the arguments above, the objectives of this study are to:

1. Study the positive effects on local economic development and identify which market niche is most suitable for the destination. On the other hand, taking into account the least negative effects on the environment and determining the most suitable market segments for the care of the environment are relevant objectives as well. Finding the target segments for the destination operator based on the combined outcome of the previous two goals would be a final conclusion.
2. Determine the effectiveness of CHAID algorithms as a method of analysis when studying the role of variables of diverse nature (travel characteristics, economic or demographic variables) in market segmentation based on the means of transportation used by Canary Islands residents and non-resident visitors.
3. Discover the characteristics of market segments associated with the use of each means of transportation as a criterion variable, basically by considering whether own vehicle, rental car or public transportation are used. This is also to identify the explanatory level of other variables, such as accommodation type, duration of visit and some other demographic variables.

Based on the objectives above and for the particular case of visitors to El Teide National Park (TNP), this research sets out to test the following specific research questions: 1) Are resident tourists the ones that generate the lowest environmental impact inside the National Park? 2) Are non-resident tourists the ones that generate the highest environmental impact inside the National Park? 3) Does the resident segment have the least positive impact on the island's economy? 4) Do non-resident tourists have the greatest positive impact on the island's economy?

Finally, the study provides empirical evidence to support the quality of the information obtained on market segmentation as a result of using this method for all the variables considered as well as outlines the overall usefulness of the analysis to understanding more closely market behavior across the tourism industry. Moreover, this study provides a clear and accurate procedure for building an extended segmentation decision tree. For that reason the results obtained will be useful for the decision making process at the level of the destination.

## 2 Literature review

There are few market segmentation studies that consider travel characteristics (accommodation, means of transportation used, duration of stay, number of visits), economic variables (season), demographic items (age, gender, educational level) and geographic ones (residents/non-residents) all at the same time. The role of residents has featured in recent segmentation studies (see for example Sánchez-Fernández et al., 2019; Stylidis, 2018; Stylidis et al., 2018) but in isolation. Where integrated studies were conducted, albeit many decades ago by LaPage (1969) and Stynes and Mahoney (1980), these studies did not clearly identify distinct tourist groups based on the variables*.*

Increasing tourist arrivals and stay place a burden on scarce resources like water and land at destinations. This is particularly true for small island destinations where there are limited resources and fragile environment (Holden, 2000; UNEP, 1999). Moreover, human perceptions concerning resources can indicate a maximum level for the number of tourists and length of stay in a destination especially as means of transportation and length of stay have a clear connection with the use and consumption of natural resources (Hughes and Furley, 1996; Odell, 1975).

In recent years, some papers have studied market segmentation differentiating domestic and international populations with some of them applying the CHAID algorithm. Therefore, when studying the diverse behaviour of segments according to geographic origin, some papers have in fact used the CHAID algorithm as the analytical method (see for example Amir, Osman, Bachok and Ibrahim, 2016; Báez-Montenegro et al., 2016; Erdilek &Wolf, 1997; McKercher and Tse, 2012). Notwithstanding, these studies failed to address tourism in some circumstances while none considered the travel characteristics or the mode of transportation used as segmentation criteria.

Interestingly, although some studies have emerged recently that connect segmetation and transportation (see for example Félix et al., 2017; Ferrer-Rossel et al., 2016; Tkaczynski and Rundle-Thiele, 2019), there remains a lack of empirical studies on transportation related to the geographic origin of tourists and travel characteristics. Some tourism studies have attempted to explore transportation choice behaviour (Robbins and Thompson, 2007), preference for mode choice (Wang & Li; 2002; Money and Crotts, 2003; Nerhagan, 2003; Koppelman and Sethi, 2005; Kim and Prideaux, 2005; Tsuar and Wu, 2005; Hess, 2007**)**, travel characteristics (Hess, 2007) as well as tourist movement patterns (McKercher and Tse, 2012). None of the above papers, however, have studied the relationship between mode of transportation and tourists' geographic origins.

CHAID algorithm was first presented by Kass (1975). It has been used in consumer research though not so much in the field of market segmentation (Haughton and Ouolabi, 1997; Levin and Zahav, 2001; Magidson, 1994; Riquier et al., 1997). Within market segmentation, researchers have focused on a priori (in origin) or post hoc (when leaving destination) analyses. Commonly, a priori and post hoc analyses have been descriptive and not based on setting a criterion variable. Using Chi-square Automatic Interaction Detection a predictive analysis is conducted to establish a criterion variable associated with the rest of the variables – the independent ones. The relationship between the criterion and the independent variables forms the segments. This allows researchers to determine the segmentation based on the criterion variable and using a range of independent variables (predictors) (Chen, 2003; Díaz-Pérez et al., 2005; Díaz-Pérez and Bethencourt-Cejas, 2016; Legohérel et al., 2015). However, before using this technique, a dependent variable (criterion) and independent ones (predictors) have to be previously selected.

CHAID analysis has several advantages over other methods of tourism market segmentation. First, Chi-square being a non-parametric statistic accepts any variable distribution form. Second, interval and nominal variables can be used as independent variables (predictors) in the model. Third, continuous variables can be used as criterion variables, as they can be dichotomized. Finally, the criterion variable can be chosen depending on the objectives of destination operators, which increases the model's efficiency.

There are some important differences when comparing criterion with non-criterion methods like cluster analysis. Despite all methods being based on variables that discriminate among segments, however, using a criterion variable has several advantages. First, relying on a set of variables and not a criterion variable may not provide significant descriptors of segments. If this happens, researchers will waste their time making an analysis. Second, CHAID, classifies new cases in the segments obtained, as it builds segments that are mutually selective, and do not overlap. Thus, each object is included in only one segment (Kass, 1980).

However, it is important to note that CHAID analysis is limited by sample size, specifically the size of the sample required per predictor variables (Chung et al., 2004; null_).

## 3 Methodology

### 3.1 Description of variables

The variables considered for the segmentation will be of diverse nature: travel characteristics (means of transportation used, accommodation, duration of stay) demographic (age, educational level, gender) economic (high/low season) and geographic variables (Canary resident or non-resident, place of residence) (see Table 1).

**Table 1:** Variables.

Variable	Categories and percentages			
Age	(1) 18–25 years	8.6	(4) 46–55	21.8
	(2) 26–35	20.6	(5) 56–65	16.7
	(3) 36–45	21.8	(6) More than 65 years	10.6
Resident (Canary Islands)	(1) Non-resident	80.6	(2) Resident	19.4
Duration of stay	(1) Less than 1 h	2.2	(4) 4–6 h	24.2
	(2) 1–2 h	14.6	(5) 6–24 h	10.6
	(3) 2–4 h	43.0	(6) More than 1 day	5.4
Educational level	(1) Basic studies	6.2	(4)Vocational training	15.0
	(2) Secondary education	7.7	(5) University Degree	54.8
	(3) Baccalaureate/A-levels	16.3		
Gender	(1) Male	53.9	(2) Female	46.1
Mean of transport (The main one used)	(1) Own car	17.7	(5) Bicycle	2.0
	(2) Rent a car	57.7	(6) Motorbike	4.0
	(3) Taxi	1.0	(7) On foot	0.5
	(4) Rental coach or shuttle	12.3	(8) Public transport	4.8
Season	(1) High	69.5	(2) Low	30.5
Accommodation	(1) Hotel with 3 stars or fewer	11.9	(7) Camping, tent	1.3
	(2) Hotel with 4 stars	27.9	(8) Family or friends house	5.5
	(3) Hotel with 5 or more stars	5.6	(9) Cruise	1.6
	(4) Apartment	18.4	(10) Own house	17.8
	(5) Rural cottage or farmhouse	3.0	(11) Others	1.9
	(6) Rented house	5.1		
Place of residence	(1) Mainland or Balearic Islands (Spain)	19.3	(6) Russia	2.8
	(2) United Kingdom	11.9	(7) Tenerife (Canary Islands)	15.8
	(3) Germany	15.3	(8) Another Canary Island	3.6
	(4) France	5.3	(9) Other country	22.4
	(5) Italy	3.5		

Based on the impact on local economic development and environment, variables and their modalities will be grouped into two major blocks: 1) variables that contribute to local economic development and 2) variables that affect the environment (see Table 2).

**Table 2:** Classification of variables/modalities according to 1. impact on the island. 2. impact on the NP.

1. Impact on the island		
Type of impact	Major impact	Minor impact
Economic positive	Hotel Accommodation (+)	Own houses, family or friends' houses (-)
	Rented accommodation (+):	
	Rural houses, apartments,	
	Car hire (+)	Own car (-)
Environmental negative	Hotel accommodation (-)	Own houses, family or friends' houses (+)
	Apartment (-)	Rural houses (rehabilitated) (+)

2. Impact on the NP		

Environmental negative	Motocycles (-)	Bicycles/walking (+)
	own/rental cars (-)	Buses/shuttles (+)
	Stay: More than a day (-)	Stay: Less than an hour (+)
	High season (-)	Low season (+)

Within the first group would be the following accommodation types for their ability to generate economic income, ordered from highest to lowest economic impacts: hotels, rental establishments, camping, tents, own houses or family or friends' houses. With respect to transportation means, the ones with the greatest economic impact per tourist would be, from greatest to least positive economic impact: rental cars, followed by buses or shuttles or rental motorbikes and finally, own cars.

The second group consists of accommodation and transportation means which have an impact on the environment. Regarding accommodation, ordered from lowest to highest impact would be own houses or houses of family and friends as accommodation with least environmental impact, since they do not require construction nor any new land use. As for transportation means, from the least to the most impact per tourist, the result is in the following order: bicycles or walking, buses or shuttles, own or rental cars and motorbikes.

Moreover, the environmental impact on the territory of the National Park itself must be considered in a special way.

### 3.2 Information collection characteristics

An empirical and quantitative study has been carried out. Teide National Park (TNP) visitors were interviewed with a structured questionnaire divided into two sections. The first section contained questions concerning the population under study, namely: nationality or habitual residence; gender, age, studies. The second section collected information related to the characteristics of the trip: duration of stay in the Teide NP, type of accommodation and means of transportation used to reach the park and inside. Moreover, to obtain exact data about the visitors of the park an ad-hoc survey was conducted.

### 3.3 Sampling technique

Surveys were made in two phases during the period of 2016–2020 using simple random probabilistic sampling. The first phase was conducted in the high season in winter and included Easter. The second was in late spring and early summer which is low season on the Canary Islands.

Considering the latest published number of visitors to the TNP 4,727,276 visitors for 2017 (ISTAC, 2020), to achieve a maximum estimation error of 3%, the necessary sample size would be 1,066 individuals. In the end, we obtained a sample of 1,411 tourists in the TNP, which is why the estimation error lowered by nearly 2% in many cases. Table 3 shows the maximum estimation error for each variable.

**Table 3:** Maximun estimation error by variable (n = 1,411 tourists of PNT with 1,394 valid cases for MEAN OF TRANSPORT).

Variable	Maximum estimation error (percent)
Age	±2.20
Canary resident or non-resident	±2.11
Duration of stay	±2.64
Educational Level	±2.64
Gender	±2.66
Mean of transport used	±2.60
Season High/Low	±2.45
Type of accommodation	±2.39
Place of residence	±2.22

The descriptive statistics shows that the 55 percent of the visitors have a university degree (Table 1). This is lower than the corresponding proportion of tourists, around 70.0 per cent in 2019 and 2018 following the Canary Islands Government Statistics (ISTAC, 2020). However as the local population is represented in the sample as well, this mismatch is reduced.

### 3.4 Statistical analysis technique

Tourism market segmentation has commonly been conducted by adopting regression methods (Fredman, 2008; Mok and Iverson, 2000; Ng and Lew, 2009). However, owing to the number of segments and qualifying variables, other multivariate analysis techniques are needed. More recent studies have used Chi-square Automatic Interaction Detection as a market segmentation method due to its greater sophistication (McCarty and Hastak, 2007) and because there is no assumption of the restrictive principal of parametric tests for predictive variables. An initial descriptive analysis was used to calculate a contingency table (crosstab analysis) of the main variables i.e., transportation means used, accommodation type and visitors' place of residence. Following this, we used the CHAID algorithm to determine the different market segment characteristics*.*

The CHAID algorithm is applied to establish a criterion variable and select a number of independent variable categories subject to the Chi-square tests being significant. Subsequently, node formation and segment configuration is carried out and finishes when no significant relationship between the criterion and explanatory variables exist. The most significant independent variable occurs in the first node. However CHAID shows greater efficiency regarding number of variables and amount of data than other non-criterion methods, such as cluster analysis (Kass, 1980).

In applying the CHAID algorithm, the rule of thumb (or stopping rule) for the growth of the tree has a very important role (Milanovic and Stamenkovic, 2016). We have considered a minimum sample size of 30 cases for the terminal nodes (final segments). As such, we assure the assumption of normality for an ANOVA procedure to compare the means of a continuous variable of interest for each segment. In addition, if the variable of interest is a categorical one, we reach a reasonable sampling size to apply a Multinomial Logistic Regression (which requires a minimum of 10 cases per independent variable in the model).

## 4 Results

**Figure 1:**
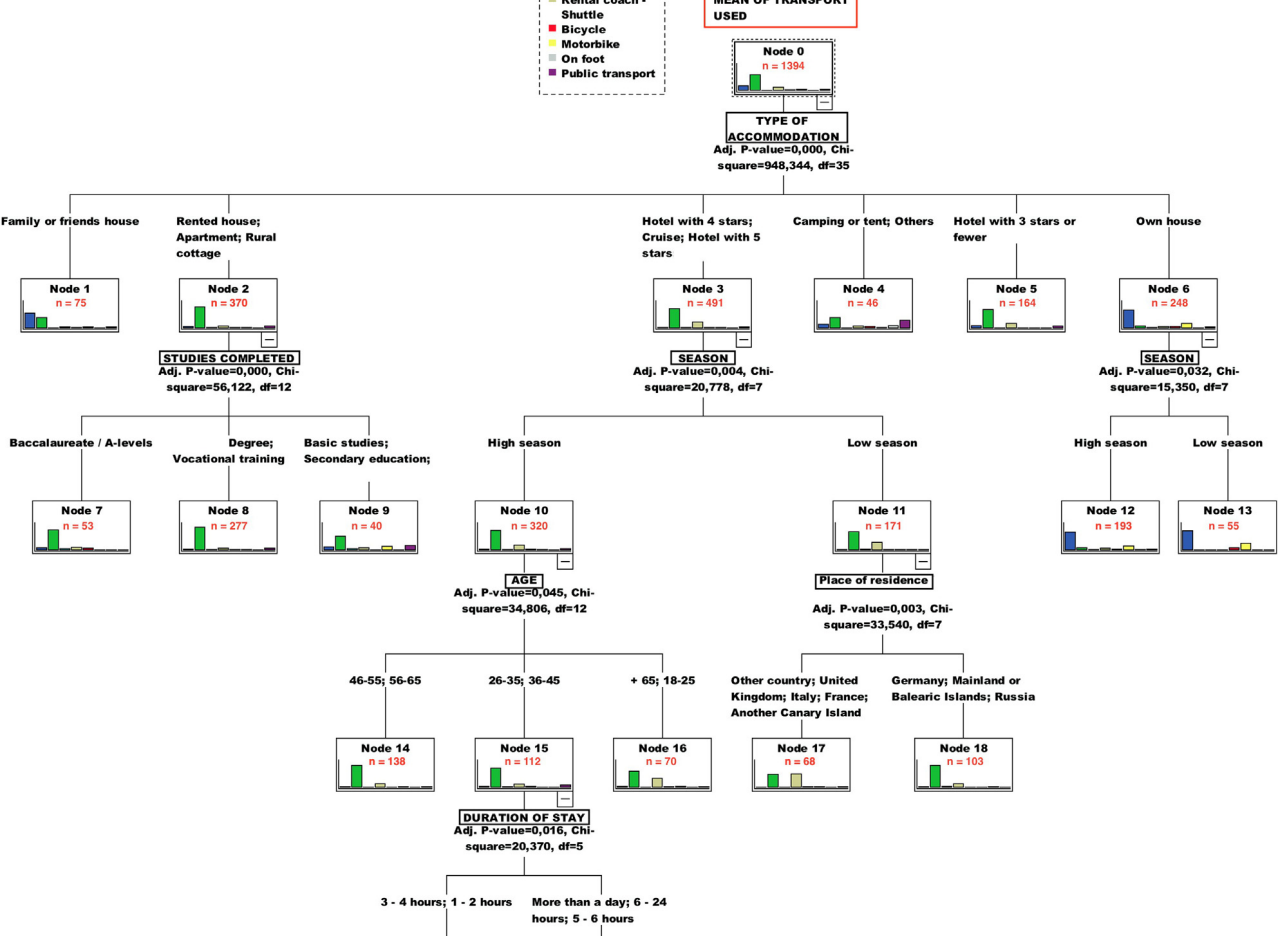
First CHAID application by default for segmenting by ‘mean of transportation used’ (nodes n > 30).

**Figure 2:**
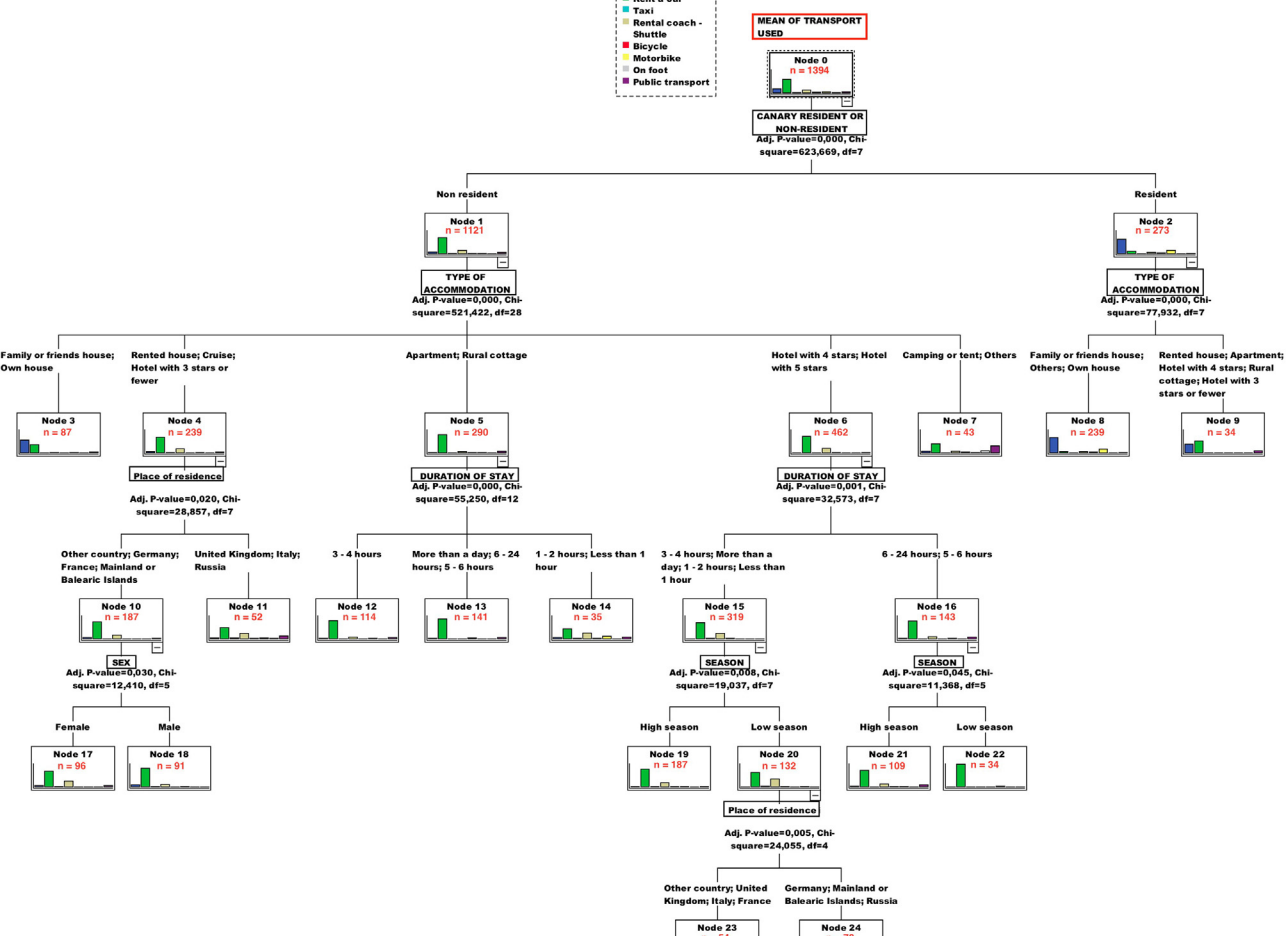
CHAID results for forcing ‘Canary residents/non-residents’ as explicative variable (nodes n > 30).

The results obtained reveal that the use of the CHAID algorithm technique shows a significant relationship when means of transportation used is chosen as a criterion variable for the classification. A decision tree is developed with four segmentation levels and 20 segments (see Figure 1). However, the Canary resident/non-resident variable does not appear explicitly in the first or any level of the classification made by CHAID, although the cross table does show a clear relationship between this variable, type of accommodation and means of transportation used. This is why we have proceeded to incorporate – to statistically force- the geographic origin of visitors, Canary resident/non-resident, in the analysis for the first node, obtaining five segmentation levels and 24 segments (see Figure 2).

### 4.1 Results relating to the application of a crosstab analysis

As a starting point in the statistical analysis, two contingency tables have been produced in which the relationships among the following variables are collected: accommodation type; means of transportation used and geographic origin of visitors. As we can observe in Table 4, the variables with the highest level of association (correlation) to MEAN OF TRANSPORT are RESIDENT/NON-RESIDENT and TYPE OF ACCOMMODATION, which justifies their more thorough analysis in the cross-reference Tables 5 and 6, which separate residents and non-residents.

**Table 4:** Significative relationship with variable MEAN OF TRANSPORT using Cramer's V for nominal variables.

1	Age	Mean of transport	V = 0.126 (p = 0.000 < 0.05)
2	Resident or non-resident	Mean of transport	V = 0.669 (p = 0.000 < 0.05), Chi-squared = 623.67
3	Duration of stay	Mean of transport	V = 0.188 (p = 0.000 < 0.05)
4	Educational level	Mean of transport	V = 0.117 (p = 0.000 < 0.05)
5	Gender	Mean of transport	V = 0.170 (p = 0.000 < 0.05)
6	Season	Mean of transport	V = 0.152 (p = 0.000 < 0.05)
7	Type of accommodation	Mean of transport	V = 0.325 (p = 0.000 < 0.05), Chi-squared = 948.34∗
8	Place of residence	Mean of transport	V = 0.275 (p = 0.000 < 0.05)

∗ First variable to appear in CHAID.

**Table 5:** Cross table Mean of Transport/Accommodation (Canary residents) (%).

Mean of transport used	Type of accommodation								Total
	Hotel 3 stars or fewer	Hotel 4 stars	Apartment	Rural cottage	Rented house	Family or friends house	Others	Own house	
Own car	1.5	1.5	1.1	0.4	0.4	4.8	1.1	51.6	62.3
Rent a car	1.8	0.7	1.5	0.4	2.2	0.4		4.4	11.4
Taxi								0.7	0.7
Rental coach - Shuttle								4.4	4.4
Bicycle						0.4		3.7	4.0
Motorbike								14.3	14.3
On foot								0.4	0.4
Public transport	0.4	0.7						1.5	2.6
Total	3.7	2.9	2.6	0.7	2.6	5.5	1.1	81.0	100.0

**Table 6:** Cross table Mean of Transport/Accommodation (Non Residents) (%).

	Hotel 3 stars or fewer	Hotel 4 stars	Hotel 5 stars	Apartment	Rural cottage	Rented house	Camping or tent	Family or friends house	Cruise	Other	Own house	
(1)	0.8	0.4	0.1	0.6	0.1	0.3	0.1	2.4	0.1	0.2	1.9	6.9
(2)	9.3	24.5	5.4	17.2	3.2	4.4	0.5	2.4	0.8	1.0	0.4	69.0
(3)	0.1	0.4		0.3		0.2			0.1			1.1
(4)	2.5	6.9	1.3	1.5	0.1	0.6		0.2	0.8	0.3		14.1
(5)	0.1	0.5	0.1	0.4	0.2		0.1			0.1		1.5
(6)	0.1	0.4		0.6		0.1		0.2	0.1	0.1		1.5
(7)	0.1	0.1					0.3			0.1		0.5
(8)	0.8	1.1	0.1	1.7		0.2	0.7	0.2		0.4	0.2	5.4
	13.8	34.2	6.9	22.3	3.6	5.7	1.7	5.4	1.9	2.1	2.4	100.0

(1) Own car (2) Rent a car.

(3) Taxi (4) Rental coach or shuttle.

(5) Bicycle (6) Motorbike.

(7) On foot (8) Public transport.

The results of the crosstab analysis appear in Table 5 (Transportation used/Accommodation for Canary residents) and 6 (Transportation used/Accommodation for Non- Canary residents).

Furthermore, we can also observe that the first variable to branch in the CHAID tree in Figure 1 will be TYPE OF ACCOMMODATION. The reason for the previous variable appearing in the first node is that, despite presenting a lower association coefficient Cramer's V (0.325) than in the case of RESIDENT/NON-RESIDENT (V = 0.669), its chi-square value is higher (948.34) (see Table 4). Therefore, considering its higher Cramer's V value, it is justified to force the RESIDENT/NON-RESIDENT variable so it can be the first in the CHAID tree.

The data collected in Table 5 clearly show that the vast majority of Teide NP visitors who live in the Canary Islands use their own car as a means of transportation (62.3%) or motorbike (14.6%) and are mainly housed in their own homes or the homes of family or friends (86.5%). That said, information contained in Table 6 relative to non-residents indicates that practically half of non-resident visitors in the Canary Islands are housed in establishments of the highest category, mainly hotels (41.1% for 4 or 5 stars) and use rental vehicles as means of transportation (83.1%), mainly rental cars, shuttle buses or coaches.

### 4.2 Results of the first CHAID application by default for segmenting by means of transportation used

The results of the CHAID application by default (see Figure 1) evidence that the most frequently used means of transportation are rental cars, own cars or coaches/shuttle buses in that order. Accommodation type is the independent variable that explains best the classification made by the CHAID to determine the segments associated with each means of transportation. The only economic significant variable in the tree is season which appears in the second level of classification with the educational level of the visitors. The age and the place of residence in groups as explanatory variables are included in the third place. Finally, duration of the stay is shown in the fourth level.

Figure 1 above and Figure 2 in the following section both include the trees obtained by establishing that terminal nodes must contain at least 30 individuals as a production criterion. The CHAID algorithm, applied to a sample of 1,394 interviewees and taking the means of transportation used to reach the park as a criterion variable, gives us a chi-square value of 948.344 with 35 degrees of freedom at the first level of segmentation, being considered significant at a value of p ≤ 0.000. As a result, in this first analysis, resident/non-resident will not be assigned by default at any node level.

### 4.3 Results of the CHAID application for forcing ‘Canary residents/non-residents’ as an explicative variable

When applying the CHAID algorithm using means of transportation used as the criterion variable, the Canary residents/non-residents variable fails to appear in the first node of the tree development (see Figure 1). At first glance, this can perhaps be explained by this technique not being suitable for segmentation vis-à-vis using this variable as a classification objective. As the previous crosstab analysis showed, there is a clear relationship between Canary residents/non-residents and the means of transportation used. This is the reason why we need to determine the circumstances that cause or generate ‘noise’ in the application of the CHAID algorithm in this particular instance. To solve this problem, therefore, it has been decided to force the variable *‘Canary residents/non-residents*’ for the first node in the CHAID analysis. The outcomes of such an intervention are shown in Figure 2. The validation of the decision tree resulting is 69.7% of correct assignations, and it has been considered as production criteria 30 individuals for parents and sons nodes.

After the variable resident/non-resident was statistically “forced”, the second decision tree (Figure 2) is obtained. This second tree opens a range of new segments for residents and non-residents which were not covered in the first one (Figure 1). The main reason for this range of new segments observed is the size of the sample. In fact, there are a total of 1,399 visitors for resident/non-resident clusters when this geographic variable has been forced to be relocated in first node.

In the particular case of residents, the segment of 273 interviews, with a Chi-square of 77.932 and 7 degrees of freedom, the decision tree is open for two new levels. The first level includes two sub-segments based on the accommodation variable, one for those visitors staying at their own, family or friends houses (239 visitors) and another for visitors staying at paid accommodation establishments (34 visitors). The first sub-segment includes resident visitors which use their own car in the vast majority of cases and the second one, visitors using rental cars in more than 50% of cases.

For the specific case of visitors staying in their own home, family or friends houses classification level, the tree demonstrates they use motorbike in a 16.3% and public transport only in a 1.7%. The final conclusion for the resident group of visitors is the following: most of them stay in their own home, with family or friends, use their own car or motorbike in some cases and do not use public transport. This result represents a qualitative advance of the CHAID segmentation in comparison to the first one contained in Figure 1. Now in Figure 2 it is possible to observe several new segments for this particular group, namely residents.

With reference to the non-resident segment, the second decision tree (Figure 2) with a sample of 1,121 interviews, a Chi-square of 521.422 and 28 degrees of freedom, includes a large amount of new segments. In fact, it is now open for four levels and a total of 20 new significant segments. The first level includes five sub-segments based on the accommodation variable: a first one for those visitors staying at their own, family or friends houses (87 visitors); a second for visitors staying at rented houses, cruise and hotel with 3 stars or fewer (239 visitors), third for visitors at apartment and rural cottage (290 visitors), fourth for visitors at hotel with 4 or 5 stars (462) and finally one for visitors at camping or tent (43 visitors). The first sub-segment includes non-resident visitors which use their own car (55.2%) and rental car (35.6%); the second one, visitors using rental car (67.8%) and bus/shuttle (18.4%); the third use basically rental car (78.6%); the fourth using rental car (72.5%) and bus/shuttle (19.9%); and finally, the fifth use rent a car (39.5%) and the public transport (30.2%). The main means of transportation used to reach the park, therefore, has been the car. The non-resident group for camping or tent is the only one which uses more environmental-friendly vehicles (public transport, for example). Considering those five sub-segments, the decision tree is open only for three of them: the second, third and fourth ones related to paid accommodation establishments. For the specific case of visitors staying in paid low category establishments, the tree is open for the duration of stay. For the 4 or 5 stars hotels is open for the economic variable, season, and for place of residence in groups (United Kingdom, Italy and France in one group and Germany, Spain's Mainland and Russia in the other). The later classification for place of residence in groups appears again for the second sub-segment, as well as gender.

The results in Figure 2 provide information about residents, of which 17.1% of the total sample are accommodated in their own houses or in houses of family or friends (see Figure 2). The previous result indicates that, if we consider the effects on the island economy, this sub-segment of residents contributes little to local development since they do not generate income from accommodation in hotels, extra-hotels, rural houses or rental homes 2.4% of the total sample. In this group, most of the visitors travel in their own cars.

Thus, according to Figure 2 residents are not the segment that contributes the most to local economic development, since they do not generate income in tourism establishments. Surprisingly, they are not the most noteworthy in terms of environmental protection either, as they mainly prefer to travel by car. Moreover, the use of cars and instead of other less polluting means of transportation, such as bicycles, buses or shuttles is detrimental from the perspective of environmental preservation.

The intermediate situation would be non-residents staying in non-hotel establishments, other than their own houses, or those of family and friends, i.e. those in rental houses, apartments, campsites, tents or cruise ships. For this second group, corresponding to Figure 2, we find that most use rental cars. Buses and shuttles are their second option and, in some cases, public transport. Thus, this second group generates more income from accommodation and car rentals and less pollution than the previous one by using buses or shuttles more often.

With regard to the non-residents listed in Figure 2, corresponding to 80.4% of the total sample, they are accommodated mainly in paid establishments (hotels of all categories, apartments, cruise, rural cottage,…). They also stand out for using buses or shuttles in the highest percentage (14.2%) of all segments, much more than residents.

The case of rural houses and apartments deserves special attention, since, although a very high percentage of these visitors use rental cars (78.6%), the establishments themselves are non-polluting and have a limited impact on the environment. This is because they mainly consist of rehabilitated houses, thus avoiding the occupation of more land. In addition, as far as economic impact is concerned, their impact is also positive, since rural houses generate income. Therefore, non-resident tourists staying in rural tourism houses generate medium economic and environmental impacts.

The final conclusion for the non-resident group of visitors is the following: most of them stay at paid accommodation establishments, use rental vehicles – bus/shuttles in some cases –and are split evenly between the number of men or women. This result represents a quantitative and qualitative advance of the CHAID algorithm approach to segmentation in comparison to the one contained in the first Figure. In the same way than for residents, in Figure 2 it is possible to observe several new segments for this particular group, namely non-residents.

### 4.4 Discussion

It is common for research on tourist market segmentation to segment the population according to demographic variables (age, gender, etc.). It is, however, much more challenging to unearth studies that combine and focus on other relevant variables such as the characteristics of a visitor's trip or economic details as well as the previously mentioned demographic ones. It is clear that, if we add the quality of our results to the above, regarding exact segments configured for individuals that are already correctly classified in segments, the results are greatly encouraging from all perspectives and especially from that of the elaboration and execution of environmental policies for tourist destinations. This is the case for this study, for which we have selected the means of transportation used by visitors to reach the TNP as a dependent variable and thus the objective of the research.

A notable contribution to be considered in this study is how it considers the use of different means of transportation, which is explained by a broad set of independent variables. This is our contribution: to explain the situation for each type (car, bus, motorcycle, etc.) based not only on the housing establishment chosen but also on whether or not the visitor is a resident on the island. Similarly, there has been no study to date that has analyzed segmentation not only based on variables but also including specific categories for each one of the following: housing establishment, length of their visit, season, and other demographic variables such as age, gender or educational level. This has all been included in a single decision tree. Moreover, the relevant finding in this study is derived from the number of segments obtained on one hand, and by the categories of the variables included in each segment on the other, which leads to numerous visitor segments with no overlap.

Although Gatta, Marcucci and Le Pira (2017) and Peeters and Schouten (2006) prove that transportation has been greatly emphasized in government planning policies, considering its major influence on any tourist destination, the thorough analysis of the various means of transportation in relation to the geographical origin of a visitor to a national park has not been performed until now. It is especially important when elaborating tourism policies to be able to segment visitors into residents and non-residents. Thus, the policymaker will undoubtedly develop different tourist policies for residents and non-residents. Nevertheless, Díaz-Pérez and Bethencourt-Cejas (2017) assert that although cross-tabulations indicated the possibility of segmenting according to geographical origin, the use of the CHAID algorithm (by default, in this case) did not allow us to identify the segments in the tree based on geographic origin.

Additionally, the number of segments obtained should be highlighted since it would be impossible to reach with other methods such as cluster analysis. The latter allows us to obtain results that can be used as general guidelines for the possible segmentation of national parks' visitor population, but it does not contribute to establishing the exact configuration of each segment with no overlap.

Moreover, the analysis conducted in this study demonstrates how the type of accommodation used by TNP visitors explains the means of transportation they used: those in their own home or that of relatives or friends uses a personal vehicle and those in hotels mainly rent a car. Since the first type of accommodation mentioned corresponds to the population of Canary Islands residents, the results above could confirm that the type of transportation used depends on whether visitors are Canary Islands residents or not. Similar results were obtained by Díaz-Pérez and Bethencourt-Cejas (2017) but, for this particular study, the CHAID algorithm did not clearly create by default a “Canary Island resident/non-resident” branch in the decision tree; in fact, if the classification methods are left working automatically, it will show some other variables in the first nodes. However, if the operating process is controlled by the researcher, very important variables from the destination management perspective may appear. This is what has happened in the study performed here: the variable in relation with the resident/non-resident character of the visitors does not appear in the classification when the first automatic application took place by default. Nevertheless, when this variable is forced for the first node of classification in the statistical analysis, the tree opens in several sub-segments, not only for non-residents but for residents as well. This is a very important result not only from a methodological point of view but also from the perspective of policymakers, whose actions may subsequently take a different course.

## 5 Conclusion & recommendations

In light of the study's findings, the results may be summarized as follows:

The analysis performed has allowed us to understand the means of transportation used by visitors according to their geographical origin as well as a set of added independent variables: accommodation establishment, length of stay, season, and other demographic variables (educational level, gender, and age). Also, more importantly, we have obtained segments with no overlap configured according to all the aforementioned variables.

Considering the economic or environmental impact, it is possible to distinguish two different categories of tourists. The first category is that of low economic impact on the island and high environmental impact on the National Park. The second category includes segments with high economic impact on the island and low environmental impact on the National Park.

Residents staying in family, friends or others' houses have a low environmental impact as they do not harm the environment by requiring the construction of new accommodations. However, they mainly use transportation with high environmental impacts such as motorbikes or their own cars. Thus, residents' environmental impact inside the National Park could be seen as high for this reason.

The second category includes segments with high economic impact on the island and low environmental impact on the National Park. Non-resident visitors staying in hotels (4 or 5∗) are an important source of economic revenues for the island as a whole. At the same time, they have an environmental impact inside the park due to the number of visitors that represent. Nevertheless, their potential environmental impact on the NP is not so high, as they use rental coaches and shuttles as a means of transportation more often than the rest of the segments.

Moving on, the CHAID algorithm analysis identifies the variable “means of transportation used” as the dependent variable of the model and, at the same time, allows the use of the “Canary Islands resident/non-resident” variable as an independent one when explaining the mode of transportation used by visitors. In fact, one of the useful conclusions to be drawn from this study is that “Canary Islands resident/non-resident” does not appear with a relevant range of segments in the tree calculated by default. To reach the aim of having important information concerning this variable, it must be forced, statistically speaking, for the first node of the decision tree. Having forced the variable resident/non-resident in the process, it is shown in the decision tree with a highly significant Chi-square. Interestingly, this is one of the most relevant advantages of CHAID algorithm segmentation in the sense that one can choose those variables most important for purposes of destination management.

Consistent with our objectives concerning environmental tourism policy, what is relevant is whether the visitors are residents or not, as these two varying segments are likely to require differentiated strategies. In turn, they will also necessitate possible political interventions with specific impacts on the approaches the destination's management makes to achieve a more holistic and sustainable development. From the perspective of environmental policies in tourism, for example, a general recommendation would be to propose a ban (or limited or monitored access) on the use of cars or motorbikes inside the park. The local population frequently visits the park by motorbike and in groups. Considering its environmental impact, the limitation in the number of vehicles in a group is therefore decisive. In addition, and because of car use frequency, it would be advisable to establish free bus routes starting from external access points to NP entrances. For the particular case of public transport, it is mandatory to connect the municipalities where the potential visitors are accommodated with the park. As the majority of the tourist accommodations are in the south of the island, regular public transport routes must be established not only from the north, but also from the south of the island.

This result showcases the importance of performing detailed analyses on the resident and non-resident character of visitors for future studies as well as the set of measures to be taken to each of the identified segments. In essence, specific analyses should be performed for visitors from different countries or regions. Thus, future studies could be related to the segmentation of non-residents according to groups of countries. The decision tree obtained here classifies tourists that visit the TNP in two country groups. As the tree stops producing new branches from this result, it would also be interesting for future studies to focus only on non-residents and with a broad enough sample that includes key markets such as visitors from the United Kingdom classified in one group and visitors from Germany in another. Also it should be considered the limitation of the educational level of the tourist population. According to the descriptive statistics analysis, 55 percent of the visitors have a university degree. Therefore and following the published statistics this percentage should be higher for data being representative in future research.

Finally, the empirical study presented here demonstrates that the CHAID algorithm is always useful as an instrument to segment tourism markets if the researcher is aware of the application rules required by this method of segmenting markets. One of the possible applications is that relevant variables could be forced by the researcher. Therefore, the researcher could force the geographic variables for those occasions when they are important from the perspective of destination management. Nonetheless, regarding future studies, cluster analyses can also be recommended for those cases where the criterion variable is not given beforehand. This is when the national park manager is not interested in knowing the reasons for visitors' behavior based on a specific variable but rather wants to have a general idea on what variables have a greater classifying capability.

## Declarations

### Author contribution statement

F.M. Diaz-Perez: Conceived and designed the experiments; Performed the experiments; Contributed reagents, materials, analysis tools or data; Wrote the paper.

C.G. García-González: Analyzed and interpreted the data; Contributed reagents, materials, analysis tools or data.

A. Fyall: Contributed reagents, materials, analysis tools or data; Wrote the paper.

### Funding statement

This work has been supported by funding from the Government of the Canary Islands and the 10.13039/501100008530European Regional Development Fund, Ref. ProID201/010125.

### Competing interest statement

The authors declare no conflict of interest.

### Additional information

Supplementary content related to this article has been published online at https://doi.org/10.6084/m9.figshare.12116385.
